# Genomic Insights into Emerging Multidrug-Resistant *Chryseobacterium indologenes* Strains: First Report from Thailand

**DOI:** 10.3390/antibiotics14080746

**Published:** 2025-07-24

**Authors:** Orathai Yinsai, Sastra Yuantrakul, Punnaporn Srisithan, Wenting Zhou, Sorawit Chittaprapan, Natthawat Intajak, Thanakorn Kruayoo, Phadungkiat Khamnoi, Siripong Tongjai, Kwanjit Duangsonk

**Affiliations:** 1 Office of Research Administration, Chiang Mai University, Chiang Mai 50200, Thailand; 2 Department of Microbiology, Faculty of Medicine, Chiang Mai University, Chiang Mai 50200, Thailand; medxchamp@gmail.com (S.Y.); punnaporn.ps@gmail.com (P.S.); wenting_z@cmu.ac.th (W.Z.); siripong.tongjai@cmu.ac.th (S.T.); 3 Guangxi Technology Innovation Cooperation Base of Prevention and Control Pathogenic Microbes with Drug Resistance, Youjiang Medical University for Nationalities, Baise 533000, China; 4 Faculty of Medicine, Chiang Mai University, Chiang Mai 50200, Thailand; sorawit1590@gmail.com (S.C.); nuttahawat@gmail.com (N.I.); thanakornkuaryoo@gmail.com (T.K.); 5 Microbiology Section, Diagnostic Laboratory, Maharaj Nakorn Chiang Mai, Chiang Mai 50200, Thailand; phadungkiat.k@cmu.ac.th

**Keywords:** *Chryseobacterium indologenes*, whole genome sequencing, nosocomial infection, emerging pathogen, extensively drug-resistant, antimicrobial resistance genes, mobile genetic elements, horizontal gene transfer

## Abstract

**Background**: *Chryseobacterium indologenes*, an environmental bacterium, is increasingly recognized as an emerging nosocomial pathogen, particularly in Asia, and is often characterized by multidrug resistance. **Objectives**: This study aimed to investigate the genomic features of clinical *C. indologenes* isolates from Maharaj Nakorn Chiang Mai Hospital, Thailand, to understand their mechanisms of multidrug resistance, virulence factors, and mobile genetic elements (MGEs). **Methods**: Twelve *C. indologenes* isolates were identified, and their antibiotic susceptibility profiles were determined. Whole genome sequencing (WGS) was performed using a hybrid approach combining Illumina short-reads and Oxford Nanopore long-reads to generate complete bacterial genomes. The hybrid assembled genomes were subsequently analyzed to detect antimicrobial resistance (AMR) genes, virulence factors, and MGEs. **Results**: *C. indologenes* isolates were primarily recovered from urine samples of hospitalized elderly male patients with underlying conditions. These isolates generally exhibited extensive drug resistance, which was subsequently explored and correlated with genomic determinants. With one exception, CMCI13 showed a lower resistance profile (Multidrug resistance, MDR). Genomic analysis revealed isolates with genome sizes of 4.83–5.00 Mb and GC content of 37.15–37.35%. Genomic characterization identified conserved resistance genes (*bla*_IND-2_, *bla*_CIA-4_, *adeF*, *vanT*, and *qacG*) and various virulence factors. Phylogenetic and pangenome analysis showed 11 isolates clustering closely with Chinese strain 3125, while one isolate (CMCI13) formed a distinct branch. Importantly, each isolate, except CMCI13, harbored a large genomic island (approximately 94–100 kb) carrying significant resistance genes (*bla*_OXA-347_, *tetX*, *aadS*, and *ermF*). The absence of this genomic island in CMCI13 correlated with its less resistant phenotype. No plasmids, integrons, or CRISPR-Cas systems were detected in any isolate. **Conclusions**: This study highlights the alarming emergence of multidrug-resistant *C. indologenes* in a hospital setting in Thailand. The genomic insights into specific resistance mechanisms, virulence factors, and potential horizontal gene transfer (HGT) events, particularly the association of a large genomic island with the XDR phenotype, underscore the critical need for continuous genomic surveillance to monitor transmission patterns and develop effective treatment strategies for this emerging pathogen.

## 1. Introduction

*Chryseobacterium indologenes* (*C. indologenes*), a non-fermenting Gram-negative bacterium, was previously classified as *Flavobacterium indologenes* [1]. It is a rare opportunistic pathogen that inhabits diverse environments, including soil, water, food, animals, and plants [2,3]. While typically non-pathogenic, *C. indologenes* has recently emerged as a significant concern in healthcare settings, particularly among immunocompromised patients, elderly individuals, and newborns [1,4]. Its ability to colonize medical equipment and survive in hospital environments such as on surfaces including hospital equipment (e.g., medical devices, dialysis machines, respiratory therapy equipment), in water supplies (e.g., tap water, showerheads), and moist environments within healthcare facilities (e.g., sinks, drains) contributes to its role as a nosocomial pathogen [5]. *C. indologenes* can cause a range of serious infections, including pneumonia, urinary tract infections (UTIs), and bloodstream infections, often associated with the use of invasive medical devices [6].

A major challenge in treating *C. indologenes* infections is the increasing prevalence of multidrug-resistant strains [7]. These strains exhibit intrinsic resistance to a broad spectrum of antibiotics, including β-lactams, due to the production of enzymes such as metallo-β-lactamases (MBLs) [8]. Furthermore, *C. indologenes* also demonstrates resistance to several other antibiotic classes, including aminoglycosides, fluoroquinolones, tetracyclines, and polymyxins [9].

*C. indologenes* possesses several virulence factors that contribute to its pathogenicity, especially in immunocompromised individuals. These include biofilm formation, which enhances resistance to antibiotics and host immune responses [10]. The production of extracellular enzymes such as proteases, lipases, and DNases facilitates tissue invasion and nutrient acquisition [11]. Additionally, the bacterium’s ability to adhere to surfaces (e.g., catheters) via fimbriae or other adhesins further promotes persistent infections and complicates treatment, increasing the risk of severe illness and potentially sepsis [12,13]. Although *C. indologenes* infections have been reported worldwide, information regarding this bacterium and its characteristics remains limited.

Advancements in genomic technologies, such as WGS, have significantly improved our understanding of pathogens [14]. This technique has been applied to *C. indologenes* in countries such as China and France to gain a deeper understanding of its genetic makeup and prevalence [15,16]. The genome of *C. indologenes* is approximately 4.8 million base pairs long and contains about 4000 genes [17]. WGS has revealed that *C. indologenes* harbors numerous genes that contribute to its virulence, including those that encode toxins, adhesins, and enzymes that facilitate immune evasion [17]. WGS has also identified the presence of numerous antibiotic resistance genes, including those encoding enzymes that inactivate various classes of antibiotics [15,17].

The comprehensive genomic data derived from WGS of *C. indologenes* is indispensable for developing more effective strategies to combat infections caused by this challenging bacterium. Despite the escalating incidence of *C. indologenes* infections, WGS studies are notably scarce, especially in Southeast Asia, including Thailand. This study, therefore, aims to meticulously characterize clinical *C. indologenes* isolates from Thailand using WGS, investigating their genetic makeup, mechanisms of antibiotic resistance, virulence potential, and mobile genetic elements. Ultimately, these findings provide actionable genomic insights to combat *C. indologenes* infections, including identifying novel drug targets, guiding combination therapies, and improving resistance surveillance. They also support the development of rapid diagnostics to refine clinical management, optimize empirical and targeted treatments, and strengthen infection control. Additionally, this knowledge enhances regional epidemiological understanding of *C. indologenes* resistance dynamics.

## 2. Results

### 2.1. The Bacteria Collection Demographic and Clinical Characteristics

In this study, a total of 12 non-duplicated *C. indologenes* isolates consisting of CMCI01, CMCI05, CMCI10, CMCI11, CMCI12, CMCI13, CMCI14, CMCI23, CMCI46, CMCI56, CMCI60, and CMCI63 were identified from clinical specimens of Maharaj Nakorn Chiang Mai Hospital, Thailand.

The demographic and clinical characteristics of the 12 patients from whom *C. indologenes* isolates were collected are summarized here. Complete clinical data were available for 10 cases (Supplementary Table S1). The cohort demonstrated a predominance of male patients (83.33%), with a mean age of 63.33 years (range: 44–82), indicating infections often occurred in elderly individuals. Most patients (75%) were hospitalized, primarily in the internal medicine wards (66.67%), with 33.33% admitted to the ICUs. Underlying medical conditions were frequently observed. Common underlying diseases included malignancy (20%), lung diseases (20%), and liver cirrhosis (10%). Additionally, various comorbidities were prevalent, such as anemia (55.55%), acute renal failure (55.55%), and essential hypertension (44.44%). Contributing clinical factors observed in this cohort included the presence of intravenous catheters (90%), urinary catheters (70%), and respirators (70%), with surgery being a preceding event in 30% of cases. Specimens were predominantly collected from urine (83.33%) and sputum (16.67%). Furthermore, some of the cases (41.67%) presented with co-infections by other pathogens, such as *Pseudomonas aeruginosa*, *Acinetobacter baumannii*, and yeast.

### 2.2. Antimicrobial Susceptibility

The minimum inhibitory concentration (MIC) values for each strain and the corresponding interpretations of their antibiotic susceptibility are presented in Table 1. Based on the results, *C. indologenes* isolates predominantly exhibited resistance to at least five of the tested antibiotics, particularly piperacillin-tazobactam, ceftriaxone, cefepime, imipenem, and meropenem, with a resistance rate of 100%. Additionally, these isolates showed high-level resistance as indicated by MIC_50_ and MIC_90_ values. The data revealed that most isolates were resistant to at least one agent in nearly all antimicrobial categories—except trimethoprim/sulfamethoxazole. Notably, only CMCI13 was resistant to at least one agent in three antimicrobial classes. Therefore, CMCI13 was identified as an MDR isolate, exhibiting a lower level of resistance compared to the others. In contrast, 11 *C. indologenes* isolates were classified as XDR (Supplementary Figure S1).

### 2.3. Whole Genome Sequences and General Characteristics of C. indologenes Genome

The whole genome sequence data of our isolates, assembled and validated using the CheckM2 tool (version 1.0.1) for quality control, have been successfully deposited in the NCBI GenBank database.

The features of the hybrid-assembled genomes of 12 *C. indologenes* isolates are presented in Table 2. The results revealed that genome sizes ranged from 4.83 to 5.00 Mb, each comprising a single circular chromosome. The genomes exhibited a G + C content of 37.17–37.35% and contained between 4344 and 4488 coding sequences (CDSs). Additionally, each genome contained 18 genes for ribosomal RNAs (rRNAs), 84–87 genes for transfer RNAs (tRNAs), and 1 gene for transfer-messenger RNA (tmRNA). Among this collection, the genome of CMCI13 was the largest, with minor differences in GC content.

### 2.4. Orthologous Genes Analysis

**Figure 1:**
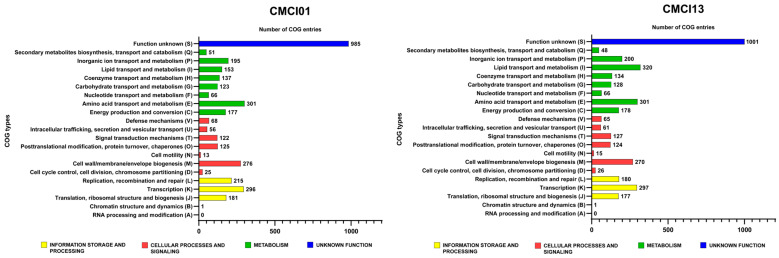
Clusters of orthologous groups (COG) analysis of *C. indologenes* strains CMCI01 and CMCI13 was performed based on functional annotation using eggNOG-mapper (version 2).

The distribution of genes involved in protein synthesis within the Cluster of Orthologous Groups (COG) of *C. indologenes* isolates was displayed in Figure 1. It was found that the total number of genes ranged from 4060 to 4211. Most of the isolates exhibited similar patterns of gene distribution, categorized into 18 COG types. The majority of genes were described as having unknown functions, followed by genes involved in amino acid metabolism and transport, transcription, and cell wall/membrane/envelope biogenesis. Additionally, genes related to replication, recombination/repair, and inorganic ion transport/metabolism were also identified. However, there was a slight difference in the strain CMCI13 compared to other strains, which showed a higher or lower proportion of gene categories.

### 2.5. SNPs in C. indologenes Genomes

Genetic variations in the genomes of 12 *C. indologenes* isolates were analyzed by identifying single-nucleotide polymorphisms (SNPs) relative to the reference genome of *C. indologenes* ATCC 29897. All isolates, obtained from Maharaj Nakorn Chiang Mai Hospital, exhibited SNP differences compared to the reference. The total number of SNPs per isolate ranged from 19,351 to 27,032. Among these, 84–91 SNPs were predicted to cause frameshift mutations, while 5451–5863 SNPs resulted in missense mutations. Additionally, 14–16 SNPs were associated with disruptive in-frame insertions or deletions, and 10–12 SNPs corresponded to conservative in-frame insertions or deletions. The quantification of other categories of SNPs is detailed in Supplementary Table S2.

### 2.6. Comparative Genome Analysis

#### 2.6.1. Phylogenetic Analysis

**Figure 2:**
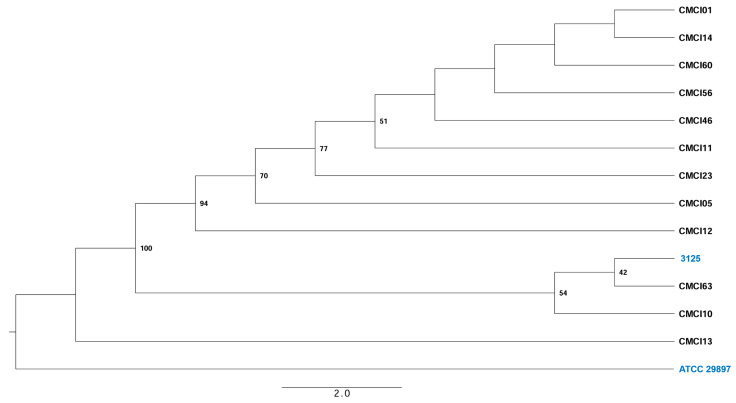
A single-nucleotide polymorphism (SNP)-based maximum-likelihood phylogenetic tree was constructed for 14 *C. indologenes* strains using the GTR + FO + ASC + R5 model to illustrate their evolutionary relationships, including isolates analyzed in this study. Bootstrap support values were calculated from 1000 replicates and are displayed at the corresponding branches. Branch lengths represent genetic distances, as indicated by the scale bar (2.0).

The evolutionary relationships among the *C. indologenes* isolates are illustrated in the phylogenetic tree (Figure 2). Based on the branching patterns, several pairs of closely related strains can be identified, including CMCI01 and CMCI14, CMCI60 and CMCI56, as well as CMCI46 and CMCI11. Notably, the clinical isolate 3125, originating from China, clusters closely with CMCI63 and CMCI10, suggesting a shared evolutionary background within this subgroup. In contrast, CMCI13 forms a distinct branch together with the reference strain ATCC 29897, indicating that CMCI13 is genetically the most divergent from the other CMCI isolates analyzed in this study. This phylogenetic pattern underscores the genetic diversity among the isolates, while also highlighting specific strain clusters that may reflect clonal relationships or geographic lineage patterns.

#### 2.6.2. Pangenome and Average Nucleotide Identity (ANI) Analyses

The pangenome analysis of 12 *C. indologenes* isolates, along with the reference strain ATCC 29897 and the Chinese clinical isolate 3125, was performed using Anvi’o (version 8) and is presented in Supplementary Figure S2. This analysis identified 5017 gene clusters (GCs), comprising 3694 core genes and 1312 accessory genes. Gene annotation revealed that most of these clusters are associated with housekeeping functions, particularly those related to translation, ribosomal structure, and biogenesis, followed by genes involved in cell wall/membrane/envelope biogenesis.

The heatmap and dendrogram derived from the Average Nucleotide Identity (ANI) analysis demonstrated that the majority of *C. indologenes* isolates—including CMCI12, CMCI05, CMCI46, CMCI60, CMCI23, CMCI11, CMCI14, CMCI01, and CMCI56—exhibited high genomic similarity, forming a distinct cluster. This suggests that these strains share a very high genome-wide similarity, exceeding 95–96% ANI, which supports their classification within the same species or clonal group. In contrast, CMCI10, CMCI63, and CI3125 clustered separately from the main group, indicating a more distant genetic relationship. Notably, both CMCI13 and the reference strain ATCC 29897 showed greater genetic divergence compared to the other strains analyzed.

#### 2.6.3. Pairwise Single Nucleotide Polymorphism (SNP) Comparison

**Figure 3:**
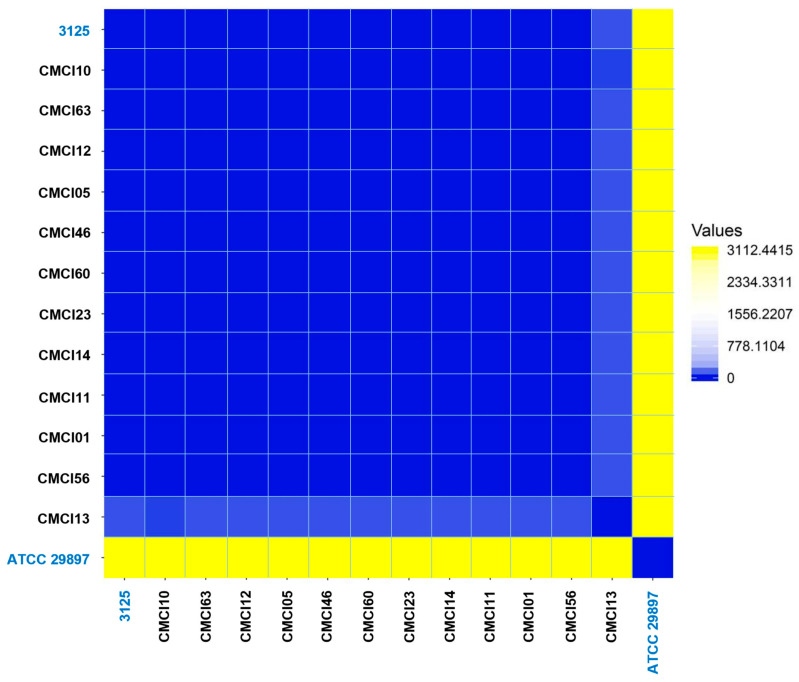
Pairwise SNP analysis of *C. indologenes* isolates. The analysis illustrates the genomic relatedness among 12 *C. indologenes* isolates in comparison with two reference strains. The heatmap visualizes the distance matrix generated from pairwise SNP comparisons using the Euclidean distance metric. Yellow indicates high SNP distance values, gradually transitioning to blue, which represents zero distance and thus high genomic similarity.

A pairwise SNP comparison, presented as a heatmap (Figure 3), revealed a predominantly tight cluster of isolates with low pairwise SNP differences. This group included eleven *C. indologenes* isolates from the collection: CMCI01, CMCI05, CMCI10, CMCI11, CMCI12, CMCI14, CMCI23, CMCI46, CMCI56, CMCI60, and CMCI63. Interestingly, the clinical isolate 3125 also clustered closely with this group, exhibiting similarly low SNP distances to all eleven isolates. In contrast, CMCI13 displayed high SNP distances when compared to both the main cluster and strain 3125, aligning more closely with the reference strain ATCC 29897.

### 2.7. Antimicrobial Resistance Gene Profiles

**Figure 4:**
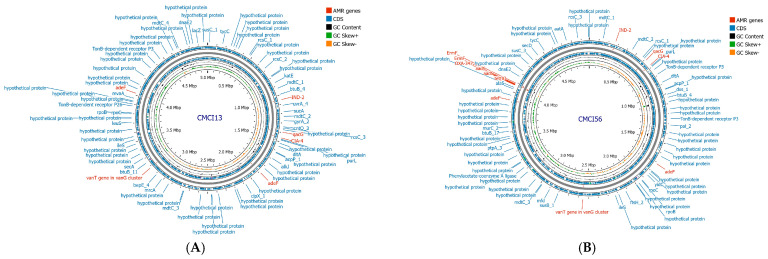
Circular genome maps of *C. indologenes* CMCI13 (**A**) and CMCI56 (**B**). Genome map visualizations were generated using Proksee (version 1.0.0a6). The maps display multiple concentric rings representing various genomic features and analyses. Predicted coding sequences (CDSs) on both the forward and reverse strands are shown in blue, with annotations of known genes and hypothetical proteins provided by Prokka v1.14.5. Genes involved in antimicrobial resistance (AMR), as predicted by CARD v4.0.1, are highlighted in red. GC content and GC skew (positive and negative) are displayed in the innermost rings of the maps.

Whole genome sequence analysis of *C. indologenes* isolates revealed the presence of antibiotic resistance genes located on the bacterial chromosome. Most isolates harbored multiple resistance genes (Table 3), including genes related to β-lactamase production (*bla*_IND_, *bla*_CIA_, *bla*_OXA_), quinolone resistance (*adeF*), tetracycline and tigecycline resistance (*tetX*), aminoglycoside resistance (*aadS*), macrolide resistance (*ermF*), and resistance to quaternary ammonium compounds (*qacG*). All *C. indologenes* isolates carried β-lactamase genes, including *bla*_IND-2_, *bla*_CIA-4_, and *bla*_OXA-347_, along with genes conferring resistance to other antibiotic classes. However, strain CMCI13 displayed a distinct resistance gene profile compared to the other isolates, lacking the *bla*_OXA-347_, *tetX*, *aadS*, and *ermF* genes. The antimicrobial resistance genes identified in the genome of CMCI13 are illustrated in Figure 4A, in comparison with another strain shown in Figure 4B.

### 2.8. Virulence Associated Gene Profiles

Prediction of virulence factors revealed the presence of various genes associated with the pathogenicity of *C. indologenes* (Table 4). Most isolates exhibited similar virulence gene profiles. These included genes related to adhesion and invasion (*pilR*, *htpB*, *fimH*, *tuf*), capsular biosynthesis (*capL*, *rmlA–C*), stress response (*katA*, *katG*), efflux pump formation (*adeG*, *mtrD*), exopolysaccharide synthesis (*pgi*), lipopolysaccharide (LPS) synthesis (*acpXL*, *rmlA*), enolase production (*eno*), and the secretion system (*clpB*, *clpV1*), among others.

### 2.9. Mobile Genetic Element Gene Profiles Within the Genome of C. indologenes

#### 2.9.1. Genomic Islands

**Figure 5:**
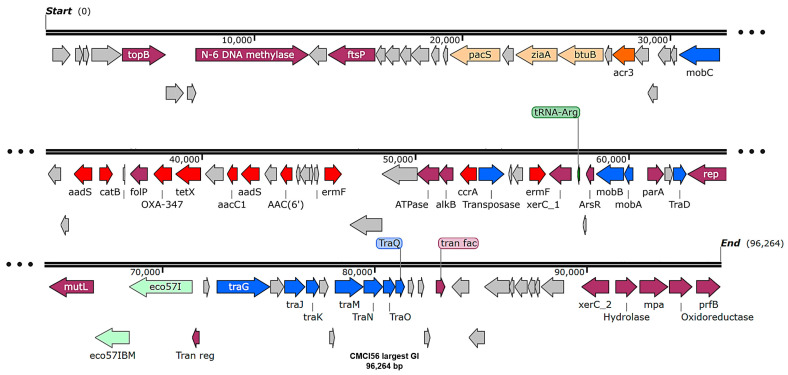
The genomic island mapping of *C. indologenes* strain CMCI56. The map was visualized using SnapGene v8.1.0. Arrows indicate predicted open reading frames (ORFs) and their transcriptional orientation. The functional categories of genes are color-coded as follows: gray represents hypothetical proteins; purple denotes genes associated with cellular functions; orange indicates transport proteins; red marks AMR genes; and blue represents conjugal transfer and transposase proteins. A *tRNA*-Arg is highlighted in green. Enzyme regions are indicated in light green.

**Figure 6:**
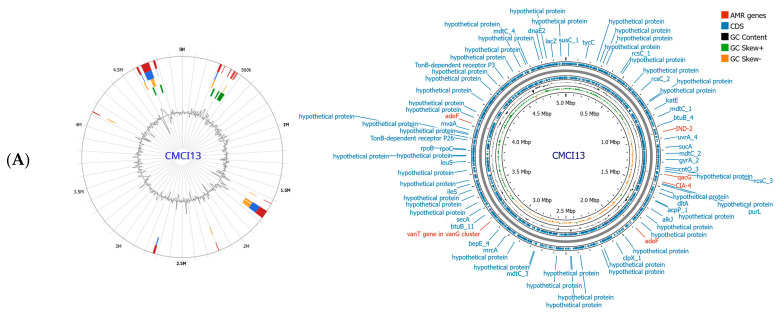

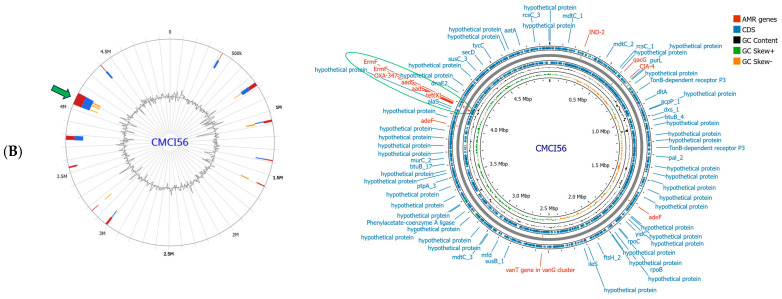
Genomic island prediction and genome map of *C. indologenes* CMCI13 (**A**) and CMCI56 (**B**). Genomic island was predicted by IslandViewer4. The colored blocks showed GIs, corresponding to the prediction method used. IslandPick (green), IslandPath-DIMOB (blue), SIGI-HMM (orange), and the integrated findings are displayed in dark red. Genome map was visualized by Proksee (version 1.0.0a6). The green arrow and green circle indicate the co-localization of the largest genomic island (GI) and AMR gene clusters.

Genomic component analysis of *C. indologenes* isolates using IslandViewer4 (webserver version 4) identified between seven and fourteen predicted GIs on each bacterial chromosome. With the exception of CMCI13, most isolates shared similar GI patterns (Supplementary Figure S3). Each isolate contained the largest GI, ranging from 94 to 100 kb (average ~96 kb) in size. Remarkably, the gene content of these largest GIs was also highly conserved. These regions predominantly harbored genes encoding hypothetical proteins, as well as genes related to essential cellular functions, transporters, integrases, and virulence factors (Figure 5). Notably, the largest GIs contained a cluster of antibiotic resistance genes, including *bla*_OXA-347_, *tetX*, *aadS*, and *ermF*. The GI patterns of isolates CMCI13 and CMCI56 (which contain a large GI) are illustrated in Figure 6A and Figure 6B, respectively.

#### 2.9.2. Insertion Sequences

The analysis of *C. indologenes* genomes using ISfinder revealed that all tested isolates possess multiple insertion sequences (ISs), as detailed in Table 5. Specifically, isolates CMCI01, CMCI05, CMCI10, CMCI11, CMCI12, CMCI14, CMCI23, CMCI46, CMCI56, CMCI60, and CMCI63 share four common IS elements, IS1272, ISElsp1, ISLpn9, and ISMsp1, originating from *Staphylococcus haemolyticus*, *Elizabethkingia* sp., *Legionella pneumophila*, and *Microscilla* sp., respectively. Notably, isolate CMCI13 also contains these four IS elements but additionally harbors ISEIan2 and ISBun1, which are associated with *Bacteroides uniformis* and *Microscilla* sp., respectively.

#### 2.9.3. Integrative Conjugative Elements

**Figure 7:**
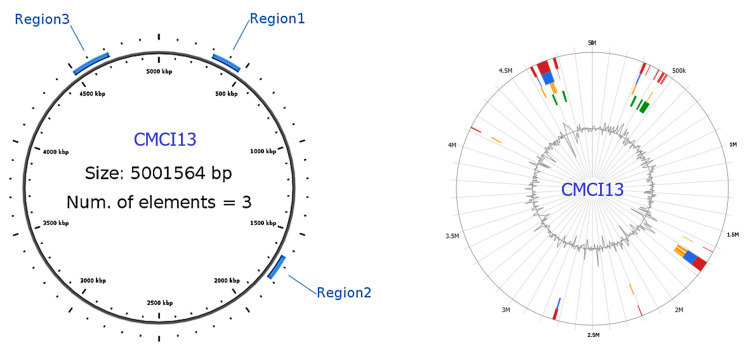
Integrative and conjugative elements and genomic islands of *C. indologenes* CMCI13 isolates are depicted. A circular diagram represents the genome of *C. indologenes* CMCI13. The blue highlights indicate the locations and sizes of three identified putative ICE regions within the genome. All ICE regions are co-located with distinct GIs.

Detection of ICEs within *C. indologenes* isolates using ICEfinder webserver (ICEberg 2.0 database) revealed that most isolates do not possess ICEs. Intriguingly, CMCI13 was the only exception, containing three distinct putative ICE regions (Figure 7). The first region spans 172,457 base pairs with a GC content of 38.69%, flanked by a 21 base pairs direct repeat (attL and attR: 5′-AATCCCTCACTCTCCGCAGAA-3′). The second region measures 149,737 base pairs with a GC content of 39.14% and is flanked by a 16 base pairs direct repeat (attL and attR: 5′-ACTTATTTTTTCTCTG-3′). The third region spans 224,937 base pairs, with a GC content of 37.48%, and is flanked by a 15 base pairs direct repeat (attL and attR: 5′-CATTGTTGTAAAAAA-3′). All three regions harbor genes associated with the type IV secretion system (T4SS), integrase, and relaxase, hallmark features of ICEs capable of T4SS-mediated horizontal gene transfer. Notably, all three ICE regions in CMCI13 co-localize with the identified GIs, suggesting a potential relationship between these MGEs.

#### 2.9.4. Integron, Plasmid, and CRISPR-Cas Analysis

The genome analysis of the *C. indologenes* 12 isolates revealed the absence of integrons, plasmid sequences, or CRISPR-Cas systems in any of the isolates.

## 3. Discussion

*Chryseobacterium indologenes* is an environmental bacterium increasingly recognized as a multidrug-resistant nosocomial pathogen, particularly affecting immunocompromised patients and newborns [1,2,3,4,5,6]. While reported globally, including in Asia [3,4,5,6], no prior studies have documented its clinical characteristics in Thailand. Whole genome sequencing is essential for understanding its genetic resistance mechanisms and virulence factors, which remain poorly characterized [12].

This study investigated 12 *C. indologenes* isolates from patients at Chiang Mai University Hospital (2021–2024), identified and confirmed via MALDI-TOF MS and 16S rRNA sequencing, respectively. The demographic and clinical characteristics of our patient cohort revealed a profile that is consistent with established risk factors for *C. indologenes* infection [1,19]. Specifically, the majority of infections occurred in elderly males (mean age 63.33 years) who had prolonged hospital stays (~18 days) and frequently presented with underlying conditions such as malignancy, chronic respiratory diseases, and liver cirrhosis. The predominant source of isolates was urine (10 out of 12 isolates), suggesting a potential uropathogenic role, while two were from sputum, indicating respiratory involvement. These observed characteristics align with previous reports from Korea and Taiwan, which also noted a predominance of male patients with comorbidities [1,19], though an Israeli study reported more female cases [20]. Given the limited number of isolates (*n* = 12), these demographic and clinical details are presented as descriptive features of our cohort, not as statistically validated risk factors.

Antimicrobial resistance profiling revealed all isolates were XDR or MDR, with universal resistance to β-lactams (including piperacillin-tazobactam, ceftazidime, ceftriaxone, and carbapenems). High resistance (>90%) was also observed for aminoglycosides (amikacin, gentamicin) and fluoroquinolones (ciprofloxacin, levofloxacin). Notably, all isolates remained susceptible to trimethoprim/sulfamethoxazole, consistent with studies from India, Taiwan, and China [21,22,23]. A single isolate (CMCI13) displayed lower resistance (MDR only), suggesting possible strain variability.

This study presents the first comprehensive genomic analysis of 12 *C. indologenes* clinical isolates from Thailand. Whole genome sequencing revealed highly conserved genome architecture, with sizes ranging from 4.83 to 5.00 Mb and GC content between 37.15% and 37.35%, consistent with previous reports of this species [15,17]. The isolates maintained stable genomic features while containing a significant proportion (approximately 30%) of genes with currently unknown functions, highlighting the need for further functional genomic studies.

Detailed SNP analysis demonstrated substantial genomic divergence from the reference strain ATCC 29897, with each isolate containing between 5451 and 5863 missense mutations [24]. This high mutational load suggests ongoing adaptive evolution in hospital environments, potentially contributing to the pathogen’s successful nosocomial persistence. Phylogenetic reconstruction revealed two distinct clusters: a predominant group of 11 isolates showing a close genetic relationship with Chinese strain 3125 [15], and a single outlier isolate (CMCI13) forming a separate branch. The pangenome analysis identified 3694 core genes involved in essential cellular processes and 1312 accessory genes that may contribute to niche adaptation.

Whole genome sequencing revealed chromosomal AMR genes in all isolates, with conserved β-lactamases *bla*_IND-2_ and *bla*_CIA-4_, matching reports from France, Taiwan, and China [16,25,26]. The *bla*_OXA-347_ variant differed from Brazil’s *bla*_OXA-209_ [17], while fluoroquinolone (*adeF*) and aminoglycoside (*aadS*) resistance genes aligned with findings from Brazil and Pakistan [17,27]. CMCI13 uniquely lacked *bla*_OXA-347_, *aadS*, *tetX*, and *ermF*, suggesting divergent evolution [28]. Eleven isolates showed broad-spectrum resistance correlating with their AMR gene profiles. Strikingly, six of nine AMR genes matched Chinese strain 3125 [15], indicating close genetic ties between Thai and Chinese isolates. These findings highlight the extensive resistance mechanisms in Thai *C. indologenes* clinical strains.

All isolates exhibited similar virulence gene profiles, including adhesion factors (*tuf*), stress response genes (*clpB*, *katA*), and efflux pumps (*adeG*, *mtrD*), which are crucial for biofilm formation, oxidative stress resistance, and immune evasion [19,29,30,31,32,33,34]. These findings align with reports from Indian *C. indologenes* strains [33], particularly the role of *tuf* in adhesion as observed in related *Chryseobacterium* species [34].

The analysis of MGEs further elucidated the potential for HGT [35]. All isolates contained GIs, which are often linked to virulence and antibiotic resistance [36]. Interestingly, all XDR strains shared a large GI (94–100 kb) containing critical genes, particularly a cluster of AMR genes. Chinese strain 3125, highly similar to our isolates, also carries a 99 kb GI with multiple AMR genes [15], strongly supporting the view that these large GIs act as key vehicles for disseminating resistance genes and driving bacterial evolutionary adaptation [37]. This mechanism parallels findings in *Klebsiella pneumoniae*, where GIs facilitate AMR spread [38]. Notably, while some AMR genes outside this large GI were conserved across all isolates, the resistance genes located within the GI (including *bla*_OXA-347_, *tetXtetX*, *aadS*, and *adeF*) were absent in the CMCI13 (MDR) strain due to the lack of a large GI. Although *C. indologenes* is intrinsically resistant to several antibiotics [38], the presence or absence of GIs carrying AMR genes in different strains may lead to phenotypic variation. Thus, the XDR/MDR phenotypes observed in this bacterial collection likely depend on acquired resistance mediated by HGT via GIs [39].

The alarming prevalence of XDR and MDR phenotypes in these *C. indologenes* isolates, directly correlated with the presence of multiple chromosomal AMR genes and a large genomic island, carries significant implications for the clinical management of infections caused by this pathogen [40]. Our genomic insights into the specific resistance determinants, such as the β-lactamases (*bla*_IND-2_, *bla*_CIA-4_, and GI-mediated *bla*_OXA-347_), provide crucial molecular understanding of the observed universal resistance to β-lactams, including carbapenems [41]. Beyond β-lactams, the high phenotypic resistance to aminoglycosides and fluoroquinolones is corroborated by the presence of genes like *aadS* and *adeF*, respectively [27]. Furthermore, the detection of *tetX* for tetracycline resistance and *ermF* for macrolide resistance [27], particularly within the identified large GI, highlights the broad spectrum of acquired resistance mechanisms present in these strains. This genetic information underscores the urgent need for genomic surveillance to rapidly identify these diverse resistance mechanisms, which can directly inform empirical antibiotic choices in clinical settings where *C. indologenes* is suspected. Notably, the consistent susceptibility to trimethoprim/sulfamethoxazole positions it as a more viable therapeutic option, contrasting sharply with the widespread resistance to multiple critical antimicrobial classes [1]. Understanding the genetic basis of virulence factors and mobile genetic elements, such as the identified genomic islands, is also critical for designing effective infection control strategies and preventing the further spread of these highly resistant strains within healthcare environments [42]. Furthermore, by correlating genotypic resistance determinants with phenotypic antimicrobial susceptibility profiles, this study contributes valuable information for clinicians in selecting effective therapeutic options against *C. indologenes*, a pathogen for which no standardized treatment guideline currently exists.

The shared presence of IS elements (IS1272, ISElsp1, ISLpn9, ISMsp1) across most isolates suggests HGT from diverse bacterial sources (*Staphylococcus* sp., *Elizabethkingia* sp., *Legionella* sp., *Microscilla* sp.), potentially mediated by IS activity [43]. CMCI13 displayed a unique IS profile containing additional ISEIan2 and ISBun1, indicating distinct HGT events that may explain its phenotypic differences [44,45]. While comparative data on IS elements in *C. indologenes* remains limited, similar IS-mediated resistance spread has been documented in other nosocomial pathogens like *Acinetobacter* sp. and *Pseudomonas* sp. [44,45].

CMCI13 uniquely harbored three ICEs co-localized with GIs, yet paradoxically showed fewer AMR genes than other isolates. This suggests these ICEs may primarily facilitate the acquisition of adaptive traits unrelated to antimicrobial resistance, consistent with observations of SXT/R391 ICEs in *Vibrio cholerae* that carry heavy metal resistance genes rather than AMR determinants [46,47]. The capacity of these ICEs to drive genomic restructuring supports findings highlighting ICEs as major contributors to bacterial genome plasticity through large-scale DNA rearrangements [48]. The dynamic nature of ICEs can lead to either gene loss through imprecise excision or new gene acquisition, underscoring their role as genetic modifiers in bacterial evolution [49,50,51,52].

In most strains of our study, the absence of ICEs and the conservation of large GIs harboring critical genes suggest that these GIs are structurally stable [53,54]. This contrasts with ICE-associated GIs, which exhibit higher genetic plasticity due to ICE-mediated excision or integration events [55,56]. The stability of ICE-free GIs may reflect strong selective pressure to maintain essential functions, whereas ICEs serve as drivers of phenotypic diversification in other contexts [57].

Notably, all 12 isolates lacked integrons, plasmids, and CRISPR-Cas systems, indicating limited reliance on these conventional MGEs for adaptation [58,59,60]. This genomic profile suggests *C. indologenes* may depend more on intrinsic resistance mechanisms and alternative pathways like GIs and IS elements or chromosomal mutations for genomic plasticity [61,62,63]. The absence of CRISPR-Cas systems could increase phage susceptibility while permitting unrestricted foreign DNA integration [64,65]. These characteristics distinguish *C. indologenes* from many Gram-negative pathogens that typically exploit MGEs for rapid adaptation [66], and may explain its comparatively lower prevalence among nosocomial bacteria [67,68].

This study offers significant genomic insights into clinical *C. indologenes* isolates in Thailand, elucidating their resistance profiles, virulence determinants, and mobile genetic elements. The discovery of a large genomic island strongly associated with the XDR phenotype, for instance, provides critical molecular targets for understanding drug resistance in this pathogen.

However, it is essential to consider the limitations of the current study. Our analysis was based on 12 isolates from a single super-tertiary referral hospital, Maharaj Nakorn Chiang Mai Hospital, which, despite its role as a major referral center for Northern Thailand, may not fully capture the complete genetic diversity or epidemiology of *C. indologenes* across the entirety of Thailand. Future research should prioritize expanding the sample size and obtaining isolates from diverse geographical locations and healthcare settings across the country. Such broader surveillance, leveraging advanced genomic techniques, will be crucial to confirm the observed genomic features, trace transmission patterns more comprehensively, and inform more generalizable treatment and infection control strategies for *C. indologenes* nationally.

Additionally, while this study provides important insights into the genomic architecture, resistance mechanisms, and virulence gene profiles of *C. indologenes*, we acknowledge that the functional annotations presented were derived from bioinformatics predictions and have not yet been confirmed through in vitro or in vivo validation. Future research should include functional validation using techniques like quantitative RT-PCR, RNA-seq, gene knockout/overexpression, infection models, and proteomics to confirm gene expression, assess their contribution to phenotype, and evaluate their in vivo relevance under relevant environmental conditions (e.g., antibiotic stress, oxidative stress).

## 4. Materials and Methods

### 4.1. Bacterial Collection, Identification, Culture, and Clinical Information

*C. indologenes* isolates were randomly collected from the Microbiology Unit, Diagnostic Laboratory, Maharaj Nakorn Chiang Mai Hospital, Chiang Mai, Thailand, between 2021 and 2024. This collection comprised clinical isolates obtained from various patient specimens. Each isolate was collected from a single patient and had to be the dominant bacterium with significant growth, excluding contaminants.

All isolates were identified by the diagnostic laboratory using mass spectrometry (MALDI Biotyper^®^, Bruker Corp., Billerica, MA, USA), with species confirmation via PCR-based 16S rRNA sequencing using the primer pair: 27F (5′-AGAGTTTGATCMTGGCZT CAG-3′); 1492R (5′-TACGGYTACCTTGTTACGACTT-3′) [69]. Bacterial isolates were cultured on Luria–Bertani (LB) agar and incubated overnight at 37 °C. Stock cultures were preserved at −80 °C in LB broth supplemented with 15% glycerol.

Additionally, demographic and clinical characteristics—including age, gender, underlying diseases, comorbidities, predisposing factors, hospitalization status, and medical wards—were recorded for the patients associated with the collected isolates.

### 4.2. Antibiotic Susceptibility

Antimicrobial susceptibility testing of *C. indologenes* was conducted using the Sensititre™ ARIS™ 2X system (Thermo Fisher Scientific, Waltham, MA, USA) to determine MICs. Twelve antibiotics were tested: piperacillin-tazobactam, ceftazidime, ceftriaxone, cefepime, imipenem, meropenem, amikacin, ciprofloxacin, levofloxacin, cefotaxime, gentamicin, and trimethoprim/sulfamethoxazole.

Testing followed the manufacturer’s protocol: a 0.5 McFarland-standardized inoculum was prepared from pure cultures, diluted in cation-adjusted Mueller–Hinton broth (CAMHB), and loaded into Sensititre GNID panels. The panels were loaded into the Sensititre ARIS 2X machine, which automatically incubated them at 35 ± 2 °C for 16–24 h and determined MICs via fluorometric detection. Results were interpreted using Sensititre SWIN™ software (version 3.3) and CLSI breakpoints (Susceptible, S; Intermediate, I; Resistant, R) [18]. Isolates resistant to at least one agent in three or more antimicrobial categories were classified as MDR, while isolates resistant to at least one drug in all but two or fewer antibiotic groups were considered XDR [70].

### 4.3. DNA Extraction, Whole Genome Sequencing, and Sequence Quality Control

Genomic DNA from *C. indologenes* was extracted using ZymoBIOMICS DNA Kits (Zymo Research Corporation™, Tustin, CA, USA) following the manufacturer’s instructions.

Whole genome sequencing was performed using a hybrid assembly approach combining Illumina short reads and Oxford Nanopore Technologies (ONT) long reads. This strategy was employed to ensure maximum accuracy and completeness of genome assemblies, particularly given the limited number of high-quality *C. indologenes* genomes currently available in public databases.

The long reads from ONT allowed us to resolve complex genomic structures and close gaps, while the high base-level accuracy of Illumina reads enabled reliable error correction. This hybrid approach provides a more accurate and complete genome assembly than either platform alone, which is particularly important for characterizing antimicrobial resistance (AMR) genes, mobile genetic elements, and SNP-based phylogenetic comparisons [71].

For Illumina sequencing, a 350 base pairs insert library was prepared via fragmentation, adapter ligation, and PCR amplification and sequenced on an Illumina NovaSeq PE150 (Illumina, San Diego, CA, USA) platform, generating 2 × 150 base pairs paired-end reads [72].

For ONT sequencing (ONT, Oxford, UK), DNA libraries were prepared using the Rapid Barcoding Kit (SQK-RBK004) without size selection and sequenced on a GridION (R9.4 flow cell) for 48 h. Basecalling was performed with Guppy v6.5.7 (using configuration dna_r9.4.1_450bps_sup.cfg). Subsequent quality filtering was performed with NanoFilt (https://github.com/wdecoster/nanofilt, accessed on 9 February 2024), demultiplexing with Guppy, and adapter trimming with Porechop [73].

### 4.4. Genome Assembly, Annotation, and Genome Submission

The genomes of each *C. indologenes* isolate were assembled using Unicycler (version 0.5.0) [74,75] with quality-controlled short-read and long-read FASTQ data. The assembly process began by constructing and scaffolding short reads into longer contigs using SPAdes (version 3.15.4) [76], which were then further assembled with long-read bridges generated by Miniasm-0.3 (r179) [77] and polished with Racon (version 1.4.3) [78]. The starting positions of complete bacterial genomes were determined based on *dnaA* or *repA* gene locations using makeblastdb and tblastn from BLAST+ (version 2.13.0) (BLAST^®^ NCBI). Genome annotation was performed using Prokka (version 1.14.5) [79] with *C. indologenes* ATCC^®^ 29897™ GenBank files as reference. The quality of all assembled genomes (completeness and contamination) was assessed using CheckM2 (version 1.0.1) [80]. All assembled genomes were submitted to the NCBI GenBank database (https://www.ncbi.nlm.nih.gov/) and assigned accession numbers.

### 4.5. Gene Ontology and Variant Calling Analysis

Gene functional characterization was performed using both COG and KEGG KOfam annotation systems [81,82]. The gene functions annotation was carried out using eggNOG-mapper version 2 (http://eggnog-mapper.embl.de/, accessed on 26 February 2024). 

Variant calling was performed to identify and characterize genetic variations in the assembled bacterial genomes using Snippy, a FreeBayes-based tool (https://github.com/tseemann/snippy, accessed on 7 March 2024). FASTA files of the assembled genomes and the GenBank annotation of the reference strain *C. indologenes* ATCC 29897 were used for variant detection and annotation. The resulting variants were visualized using Integrative Genomics Viewer (IGV) version 3.4.1 [83]. This analysis revealed isolate-specific variants that may affect protein function and influence phenotypic traits.

### 4.6. Comparative Phylogenetic Analysis, Pangenome Analysis, and Pairwise SNPs Analysis

SNPs were extracted from the multiple sequence alignment of the core genomes of 14 *C. indologenes* strains. A SNP-based maximum likelihood phylogenetic tree was constructed using IQ-TREE v2.2.0 [84], employing ModelFinder Plus (-m MFP) to select the optimal substitution model [85]. Tree robustness was assessed with 1000 ultrafast bootstrap replicates (-bb 1000), and branch lengths were calculated using the -wbtl option.

Pangenome analysis was performed using Anvi’o version 8 [86], incorporating the reference genomes *C. indologenes* ATCC 29897 and strain 3125 for comparative genomics. Genome annotation included open reading frame prediction with Prodigal v2.6.3 [87], rRNA identification using Hidden Markov Models [88], and tRNA/single-copy gene detection based on GTDB-derived databases. Functional characterization was carried out using both COG and KEGG KOfam annotation systems [81,82]. Comparative genomic analysis was conducted with PyANI to identify shared and unique gene clusters among the isolates.

For high-resolution strain typing, pairwise SNP analysis was performed exclusively on core genes with strict 1:1 copy number ratios. SNP alignments were analyzed using Pairsnp (https://github.com/gtonkinhill/pairsnp, accessed on 21 April 2024) to calculate genetic similarity, which was subsequently converted into a Euclidean distance matrix for quantitative comparison. The resulting genetic distances were visualized as a heatmap [89], enabling clear discrimination between closely related strains. This integrated multi-omics approach provided a comprehensive characterization of both evolutionary relationships and functional genomic features.

### 4.7. Identification of Antimicrobial Resistance and Virulence-Associated Genes

Protein coding sequences were annotated using the Comprehensive Antibiotic Resistance Database (CARD) version 4.0.1: (https://card.mcmaster.ca/live, accessed on 8 May 2024) [90], ResFinder 4.1 (https://cge.food.dtu.dk/services/ResFinder/, accessed on 15 May 2024) [91,92], and Arg-annot V6 (https://pubmed.ncbi.nlm.nih.gov/24145532/, accessed on 20 May 2024) [93] databases with default parameters. The identified antibiotic resistance genes were verified through BLAST searches using an e-value cutoff of 1 × 10^5^. The AMR genes within the genome region were visualized using Proksee (version 1.0.0a6), (https://proksee.ca/, 27 May 2024) [94]. Virulence factor genes were analyzed using the VFDB database v6.0 (http://www.mgc.ac.cn/cgi-bin/VFs/v5/main.cgi, accessed on 2 June 2024) [95] with BLAST searches at an e-value threshold of 1 × 10^5^.

### 4.8. Identification of Genomic Islands, Insertion Sequences, and Other Mobile Genetic Elements

The important MGEs, including GIs, ISs, ICEs, integrons, and plasmids, were systematically identified in the *C. indologenes* genomes. Genomic islands were detected using the online tool IslandViewer4 (webserver version 4) (https://www.pathogenomics.sfu.ca/islandviewer, accessed on 10 July 2024) [96], which integrates multiple prediction methods to provide comprehensive identification and visualization of GIs. Insertion sequences were identified using ISfinder webserver (https://www-is.biotoul.fr/, accessed on 18 August 2024) [61], a database and analysis tool for bacterial insertion sequences, which allows for the detection and classification of known IS elements. Integrative Conjugative Elements were identified using ICEfinder online tool from the ICEberg 2.0 database (https://bioinfo-mml.sjtu.edu.cn/ICEfinder/ICEfinder.html, accessed on 28 December 2024). Integrons were identified using Integron Finder (version 2.0.5 + galaxy0) via the Galaxy platform (https://usegalaxy.org/?tool_id=toolshed.g2.bx.psu.edu/repos/iuc/integron_finder/integron_finder/2.0.5+galaxy0&version=latest, accessed on 23 February 2025) [97], which detects and characterizes integron structures including classes, arrays of gene cassettes, and associated mobile elements. PlasmidFinder (Galaxy Version 2.1.6 + galaxy1) (https://usegalaxy.eu/?tool_id=toolshed.g2.bx.psu.edu%2Frepos%2Fiuc%2Fplasmidfinder%2Fplasmidfinder%2F2.1.6%20galaxy1&version=2.1.6%20galaxy1, accessed on 27 March 2025) [98] was used to identify plasmids carrying antibiotic resistance or virulence genes by analyzing DNA sequences and classifying plasmids based on their replicons and other characteristic features.

## 5. Conclusions

This study represents the first genomic characterization of *Chryseobacterium indologenes* clinical isolates in Thailand, revealing a highly multidrug-resistant nosocomial pathogen primarily affecting elderly male patients with underlying conditions. Most isolates exhibited resistance to β-lactams, aminoglycosides, and fluoroquinolones, while the remaining were susceptible to trimethoprim/sulfamethoxazole. Genomic analysis identified conserved resistance genes (*bla*_IND-2_, *bla*_CIA-4_, *adeF*, *vanT*, and *qacG*) and virulence factors (adhesion, stress response, efflux pumps), with a notable absence of plasmids and CRISPR-Cas systems. A large GI carrying multiple AMR genes was found in all isolates except CMCI13, which displayed a divergent genomic profile, suggesting distinct evolutionary pathways. The presence of mobile genetic elements (GIs, IS elements, ICEs) highlights potential HGT mechanisms. These findings underscore the need for enhanced surveillance and tailored treatment strategies to combat *C. indologenes* infections in hospital settings.

## Figures and Tables

**Table 1:** Antibiotic resistance of 12 *C. indologenes* isolates.

Antibiotic	MIC Range	MIC_50_	MIC_90_	No. of Isolate		
				S (%)	I (%)	R (%)
Piperacillin/Tazobactam	>64–>64	>64	>64	0	0	12 (100)
Ceftazidime	>32–>32	>32	>32	0	1 (8.33)	11 (91.67)
Ceftriaxone	>32–>32	>32	>32	0	1 (8.33)	11 (91.67)
Cefotaxime	>32–>32	>32	>32	0	0	12 (100)
Cefepime	>32–>32	>32	>32	0	0	12 (100)
Imipenem	>16–>16	>16	>16	0	0	12 (100)
Meropenem	>16–>16	>16	>16	0	0	12 (100)
Amikacin	8–>32	>32	>32	1 (8.33)	1 (8.33)	10 (83.33)
Gentamicin	8–>8	>8	>8	0	1 (8.33)	11 (91.67)
Ciprofloxacin	1–>2	>2	>2	1 (8.33)	0	11 (91.67)
Levofloxacin	0.5–>8	>8	>8	1 (8.33)	0	11 (91.67)
Trimethoprim/sulfamethoxazole	≤1–≤1	≤1	≤1	12 (100)	0	0

MIC, A minimal inhibitory concentration value used breakpoint establishing by CLSI for non-Enterobacterales, document M100 34th edition [18]; MIC range, A minimal inhibitory concentration from the lowest value to highest value; MIC_50_, A minimum concentration value at which 50% of the isolates were inhibited; MIC_90_, A minimum concentration value at which 90% of the isolates were inhibited.

**Table 2:** Genomic characteristics of 12 *C. indologenes* isolates.

Isolate Name	Accession No.	Total Sequence Length (bp)	CDS	GC Content (%)	No. of rRNA	No. of tRNA	No. of tmRNA
CMCI01	CP187257.1	4,976,911	4474	37.17	18	86	1
CMCI05	CP187307.1	4,951,596	4344	37.17	18	87	1
CMCI10	CP192273.1	4,958,362	4338	37.18	18	86	1
CMCI11	CP192274.1	4,976,633	4362	37.17	18	87	1
CMCI12	CP192275.1	4,831,670	4232	37.16	18	84	1
CMCI13	CP192276.1	5,001,564	4411	37.35	18	88	1
CMCI14	CP192277.1	4,975,598	4359	37.17	18	87	1
CMCI23	CP192278.1	4,978,254	4359	37.17	18	86	1
CMCI46	CP192279.1	4,962,489	4472	37.18	18	88	1
CMCI56	CP192280.1	4,980,883	4480	37.17	18	88	1
CMCI60	CP192281.1	4,980,418	4488	37.15	18	86	1
CMCI63	CP192282.1	4,951,582	4453	37.20	18	87	1

Accession No., GenBank/NCBI accession number for the assembled genome sequence; total sequence length (bp): the total length of the assembled genome in base pairs; CDS, number of coding DNA sequences (protein-coding genes) predicted in the genome.; GC content (%), the percentage of guanine and cytosine bases in the genome; No. of rRNA, number of ribosomal RNA genes predicted in the genome.; No. of tRNA, number of transfer RNA genes predicted in the genome.; No. of tmRNA, number of transfer-messenger RNA genes predicted in the genome.

**Table 3:** Detection of antibiotic resistance genes in 12 *C. indologenes* isolates.

Antibiotic Class	Putative Antibiotic Resistance Genes	Isolates											
		CMCI01	CMCI05	CMCI10	CMCI11	CMCI12	CMCI13	CMCI14	CMCI23	CMCI46	CMCI56	CMCI60	CMCI63
Carbapenem	*bla*_IND_	*bla*_IND-2_	*bla*_IND-2_	*bla*_IND-2_	*bla*_IND-2_	*bla*_IND-2_	*bla*_IND-2_	*bla*_IND-2_	*bla*_IND-2_	*bla*_IND-2_	*bla*_IND-2_	*bla*_IND-2_	*bla*_IND-2_
Penicillin beta-lactam	*bla*_OXA_	OXA-347	OXA-347	OXA-347	OXA-347	OXA-347	-	OXA-347	OXA-347	OXA-347	OXA-347	OXA-347	OXA-347
Carbapenem, cephalosporin	*bla*_CIA_	CIA-4	CIA-4	CIA-4	CIA-4	CIA-4	CIA-4	CIA-4	CIA-4	CIA-4	CIA-4	CIA-4	CIA-4
Macrolide	*erm*	*ermF* (2) *	*ermF* (2)	*ermF* (2)	*ermF* (2)	*ermF* (2)	-	*ermF* (2)	*ermF* (2)	*ermF* (2)	*ermF* (2)	*ermF*	*ermF* (2)
Aminoglycosides	*aad*	*aadS* (2)	*aadS* (2)	*aadS* (2)	*aadS* (2)	*aadS* (2)	-	*aadS* (2)	*aadS* (2)	*aadS* (2)	*aadS* (2)	*aadS* (2)	*aadS* (2)
Quinolone	*ade*	*adeF* (2)	*adeF* (2)	*adeF* (2)	*adeF* (2)	*adeF* (2)	*adeF* (2)	*adeF* (2)	*adeF* (2)	*adeF* (2)	*adeF* (2)	*adeF* (2)	*adeF* (2)
Quaternary ammonium compounds	*qac*	*qacG*	*qacG*	*qacG*	*qacG*	*qacG*	*qacG*	*qacG*	*qacG*	*qacG*	*qacG*	*qacG*	*qacG*
Tetracycline	*tet*	*tetX*	*tetX*	*tetX*	*tetX*	*tetX*	-	*tetX*	*tetX*	*tetX*	*tetX*	*tetX*	*tetX*
Vancomycin	*vanT* gene in *vanG* cluster	*vanT*	*vanT*	*vanT*	*vanT*	*vanT*	*vanT*	*vanT*	*vanT*	*vanT*	*vanT*	*vanT*	*vanT*
Total genes		9	9	9	9	9	5	9	9	9	9	9	9
Total positions		12	12	12	12	12	6	12	12	12	12	11	12

* Number of gene position in each chromosome.

**Table 4:** Detection of virulence-associated genes in 12 *C. indologenes* isolates.

VF Class	Virulence Factors	Putative Genes	Reference Organisms
Adherence and invasion	Type IV pili biosynthesis	*pilR*	-
	Hsp60	*htpB*	*Legionella* sp.
	Polar flagella	*flmH*	*Aeromonas* sp.
	Elongation Factor-Tu	*tuf*	*Francisella* sp.
Colonization and immune evasion	Capsular polysaccharide	*rmlA*, *rmlB*, *rmlC*, *wcaJ*, *capL*, *epsH*	*Vibrio* sp., *Staphylococcus* sp., *Streptococcus* sp.
	Capsule biosynthesis and transport	*glf*	*Campylobacter* sp.
	Exopolysaccharide	*galE*, *pgi*	*Haemophilus* sp.
Enzyme	Hemolytic phospholipase C	*plcH*	-
	Enolase	*eno*	*Streptococcus* sp.
Iron uptake	Pyoverdine	*pvdD*, *pvdI*	-
	Heme biosynthesis	*hemB*, *hemL*	*Haemophilus* sp.
Secretion system	Hcp secretion island-1 encoded type VI secretion system (H-T6SS)	*clpV1*	-
	T6SS-II	*clpB*	*Klebsiella* sp.
Acid resistance	Urease	*ureB*, *ureG*	*Helicobacter* sp.
Endotoxin and serum resistance	LPS	*acpXL*	*Brucella* sp.
	LPS O-antigen	*Undetermined*	*Pseudomonas aeruginosa*
	LPS rfb locus	*rmlA*	*Klebsiella* sp.
Lipid and fatty acid metabolism	Isocitrate lyase	*icl*	*Mycobacterium* sp.
	Pantothenate synthesis	*panD*	*Mycobacterium* sp.
Stress adaptation	Catalase–peroxidase	*katG*	*Mycobacterium* sp.
	Catalase	*katA*	*Neisseria* sp.

VF = Virulence factor.

**Table 5:** Identification of insertion sequences in 12 *C. indologenes* genomes.

Strain	Sequences Producing Significant Alignments	IS Family	Group	Origin	Score (bits) *	E-Value **
CMCI01, CMCI05, CMCI10, CMCI11, CMCI12, CMCI14, CMCI23, CMCI46, CMCI56, CMCI60, CMCI63	IS1272	IS1182	-	*Staphylococcus haemolyticus*	61.9	9 × 10^−6^
	ISElsp1	IS3	IS3	*Elizabethkingia* sp.	56.0	6 × 10^−4^
	ISLpn9	IS4	IS10	*Legionella pneumophila*	56.0	6 × 10^−4^
	ISMsp1	IS1182		*Microscilla* sp.	54.0	0.002
CMCI13	ISEIan2	IS256	-	*Elisabethkingia anophelis*	1713.0	0.0
	IS1272	IS1182	-	*Staphylococcus haemolyticus*	61.9	1 × 10^−5^
	ISElsp1	IS3	IS3	*Elisabethkingia* sp.	56.0	6 × 10^−4^
	ISLpn9	IS4	IS10	*Legionella pneumophila*	56.0	6 × 10^−4^
	ISBun1	IS1595	ISNwI1	*Bacteroides uniformis*	56.0	6 × 10^−4^
	ISMsp1	IS1182	-	*Microscilla* sp.	54.0	0.002

The consistently high bit scores (*) and low E-values (**) observed for these alignments indicate strong and statistically significant matches, confirming the presence and identity of the insertion sequences across the analyzed *C. indologenes* isolates.

## Data Availability

The complete genome sequences of all 12 *C. indologenes* isolates have been deposited in the NCBI GenBank database under BioProject accession number PRJNA1247737. Strain designations, corresponding BioSample numbers, and GenBank accession numbers as follows: CMCI01 (SAMN47828610, CP187257.1); CMCI05 (SAMN47828650, CP187307.1); CMCI10 (SAMN48554322, CP192273.1); CMCI11 (SAMN48554324, CP192274.1); CMCI12 (SAMN48554325, CP192275.1); CMCI13 (SAMN48554399, CP192276.1); CMCI14 (SAMN48554400, CP192277.1); CMCI23 (SAMN48554413, CP192278.1); CMCI46 (SAMN48554511, CP192279.1); CMCI56 (SAMN48554512, CP192280.1); CMCI60 (SAMN48554513, CP192281.1); CMCI63 (SAMN48554514, CP192282.1). Genome data of reference genome used for genome comparison: *C. indologenes* ATCC29897 and *C. indologenes* 3125 (Bioproject, PRJNA689338; Biosample SAMN17206307; accession numbers, CP067044.1).

## Supplementary Materials

The following supporting information can be downloaded at: https://www.mdpi.com/article/10.3390/antibiotics14080746/s1, Table S1: Demographic and clinical characteristics of *C. indologenes* infection; Figure S1: Antibiotic susceptibility and interpretation of 12 *C. indologenes* isolates; Table S2: SNPs variances of 12 *C. indologenes* isolates; Figure S2: Pangenome analysis of *C. indologenes* isolates.; Figure S3: Comparison of genomic island patterns within 12 *C. indologenes* chromosomes.

## References

[1] Chang J., Kim S., Kwak Y.G., Um T.H., Cho C.R., Song J.E. (2023). Clinical and Microbiological Characteristics of *Chryseobacterium indologenes* Bacteremia: A 20-Year Experience in a Single University Hospital. Infect. Chemother..

[2] McKew G. (2014). Severe sepsis due to *Chryseobacterium indologenes* in an immunocompetent adventure traveler. J. Clin. Microbiol..

[3] Lim W.G., Tong T., Chew J. (2020). *Chryseobacterium indologenes* and *Chryseobacterium gleum* interact and multiply intracellularly in *Acanthamoeba castellanii*. Exp. Parasitol..

[4] Mehta R., Pathak A. (2018). Emerging *Chryseobacterium indologenes* Infection in Indian Neonatal Intensive Care Units: A Case Report. Antibiotics.

[5] Nemli S.A., Demirdal T., Ural S. (2015). A Case of Healthcare Associated Pneumonia Caused by *Chryseobacterium indologenes* in an Immunocompetent Patient. Case Rep. Infect. Dis..

[6] Yadav V.S., Das B.K., Gautam H., Sood S., Kapil A., Mohapatra S. (2018). *Chryseobacterium indologenes*: An emerging uropathogen among hematological malignancy patients. S. Asian J. Cancer.

[7] Parajuli R., Limbu T., Chaudhary R., Gautam K., Dahal P. (2023). Phenotypical Detection of β-Lactamases in a Multidrug-Resistant and Extensively Drug-Resistant *Chryseobacterium indologenes*: A Rare Human Pathogen With Special References to Risk Factor. Microbiol. Insights.

[8] Zeba B., De Luca F., Dubus A., Delmarcelle M., Simporé J., Nacoulma O.G., Rossolini G.M., Frère J.M., Docquier J.D. (2009). IND-6, a highly divergent IND-type metallo-β-lactamase from *Chryseobacterium indologenes* strain 597 isolated in Burkina Faso. Antimicrob. Agents Chemother..

[9] Mazzola V.C., Bono E., Pipitò L. (2025). A case of hospital-acquired pneumonia associated with *Chryseobacterium indologenes* infection in a patient with HIV infection and review of the literature. AIDS Res. Ther..

[10] Rather M.A., Gupta K., Mandal M. (2021). Microbial biofilm: Formation, architecture, antibiotic resistance, and control strategies. Braz. J. Microbiol..

[11] Mwanza E.P., Hugo A., Charimba G., Hugo C.J. (2022). Pathogenic Potential and Control of *Chryseobacterium* Species from Clinical, Fish, Food and Environmental Sources. Microorganisms.

[12] Agarwal S., Kakati B., Khanduri S. (2018). Severe Sepsis Due to *Chryseobacterium indologenes*, a Possible Emergent Multidrug-Resistant Organism in Intensive Care Unit-Acquired Infections. Indian J. Crit. Care Med..

[13] Geremia N., Marino A., De Vito A., Giovagnorio F., Stracquadanio S., Colpani A., Di Bella S., Madeddu G., Parisi S.G., Stefani S. (2025). Rare or Unusual Non-Fermenting Gram-Negative Bacteria: Therapeutic Approach and Antibiotic Treatment Options. Antibiotics.

[14] Lu Y., Li M., Gao Z., Ma H., Chong Y., Hong J., Wu J., Wu D., Xi D., Deng W. (2025). Advances in Whole Genome Sequencing: Methods, Tools, and Applications in Population Genomics. Int. J. Mol. Sci..

[15] Luo Y., Chen M., Jiang Y., Wang W., Wang H., Deng L., Zhao Z. (2023). Study on the Genome and Mechanism of Tigecycline Resistance of a Clinical *Chryseobacterium indologenes* Strain. Microb. Drug Resist..

[16] Cimmino T., Rolain J.M. (2016). Whole genome sequencing for deciphering the resistome of *Chryseobacterium indologenes*, an emerging multidrug-resistant bacterium isolated from a cystic fibrosis patient in Marseille, France. New Microbes New Infect..

[17] Damas M.S.F., Ferreira R.L., Campanini E.B., Soares G.G., Campos L.C., Laprega P.M., Soares da Costa A., Freire C.C.M., Pitondo-Silva A., Cerdeira L.T. (2022). Whole genome sequencing of the multidrug-resistant *Chryseobacterium indologenes* isolated from a patient in Brazil. Front. Med..

[18] CLSI (2024). Performance Standards for Antimicrobial Susceptibility Testing.

[19] Lin Y.T., Jeng Y.Y., Lin M.L., Yu K.W., Wang F.D., Liu C.Y. (2010). Clinical and microbiological characteristics of *Chryseobacterium indologenes* bacteremia. J. Microbiol. Immunol. Infect..

[20] Alon D., Karniel E., Zohar I., Stein G.Y. (2018). *Chryseobacterium indologenes* Bacteremia: Clinical and Microbiological Characteristics of an Emerging Infection. Int. J. Clin. Med..

[21] Jain V., Sahu C., Afzal Hussain N.A.F., Ghar M., Prasad K.N. (2018). The Era of Device Colonizers: *Chryseobacterium indologenes* Infections from a Tertiary Care Center in North India. Indian J. Crit. Care Med..

[22] Chen F.L., Wang G.C., Teng S.O., Ou T.Y., Yu F.L., Lee W.S. (2013). Clinical and epidemiological features of *Chryseobacterium indologenes* infections: Analysis of 215 cases. J. Microbiol. Immunol. Infect..

[23] Zhang Y., Zhao X., Xu S., Li Y. (2022). Clinical Characteristics and Risk Factors for Intra-Abdominal Infection with *Chryseobacterium indologenes* after Orthotopic Liver Transplantation. Pathogens.

[24] Lindstrom S., Schumacher F., Siddiq A. (2011). Characterizing associations and SNP-environment interactions for GWAS-identified prostate cancer risk markers--results from BPC3. PLoS ONE.

[25] Yeh T.K., Li Z.H., Huang Y.T., Liu P.Y. (2022). COVID-19 Associated Bacteremia with *Chryseobacterium indologenes* Co-Harboring *blaIND-2*, *blaCIA* and *blaCcrA*. Infect. Drug Resist..

[26] Lin X.H., Xu Y.H., Cheng J., Li T., Wang Z.X. (2008). Heterogeneity of *bla*IND metallo-β-lactamase-producing *Chryseobacterium indologenes* isolates detected in Hefei, China. Int. J. Antimicrob. Agents.

[27] Irfan M., Tariq M., Basharat Z. (2023). Genomic analysis of *Chryseobacterium indologenes* and conformational dynamics of the selected DD-peptidase. Res. Microbiol..

[28] Baquero F., Martínez J.L., Lanza F.V. (2021). Evolutionary Pathways and Trajectories in Antibiotic Resistance. Clin. Microbiol. Rev..

[29] Xiao L., Pu Y., Cui Y. (2025). Elongation factor Tu promotes the onset of periodontitis through mediating bacteria adhesion. npj Biofilms Microbiomes.

[30] Sivaranjani M., Leskinen K., Aravindraja C. (2019). Deciphering the Antibacterial Mode of Action of Alpha-Mangostin on *Staphylococcus epidermidis* RP62A Through an Integrated Transcriptomic and Proteomic Approach. Front. Microbiol..

[31] McCormick L.A., Mertz S.B., Park C., Wise J.G. (2021). Transport Dynamics of MtrD: An RND Multidrug Efflux Pump from *Neisseria gonorrhoeae*. Biochemistry.

[32] bd El-Rahman O.A., Rasslan F., Hassan S.S., Ashour H.M., Wasfi R. (2023). The RND Efflux Pump Gene Expression in the Biofilm Formation of *Acinetobacter baumannii*. Antibiotics.

[33] Sinha S., Aggarwal S., Singh D.V. (2024). Efflux pumps: Gatekeepers of antibiotic resistance in *Staphylococcus aureus* biofilms. Microb. Cell.

[34] Zhang Z., Yang L.L., Li C.J., Jiang X.W., Zhi X.Y. (2021). *Chryseobacterium paridis* sp. nov., an endophytic bacterial species isolated from the root of *Paris polyphylla* Smith var. yunnanensis. Arch. Microbiol..

[35] Tokuda M., Shintani M. (2024). Microbial evolution through horizontal gene transfer by mobile genetic elements. Microb. Biotechnol..

[36] Rao R.T., Sharma S., Sivakumar N., Jayakumar K. (2020). Genomic islands and the evolution of livestock-associated *Staphylococcus aureus* genomes. Biosci. Rep..

[37] Ramamurthy T., Ghosh A., Chowdhury G., Mukhopadhyay A.K., Dutta S., Miyoshi S.I. (2022). Deciphering the genetic network and programmed regulation of antimicrobial resistance in bacterial pathogens. Front. Cell. Infect. Microbiol..

[38] Muraya A., Kyany’a C., Kiyaga S., Smith H.J., Kibet C., Martin M.J., Kimani J., Musila L. (2022). Antimicrobial Resistance and Virulence Characteristics of *Klebsiella pneumoniae* Isolates in Kenya by Whole-Genome Sequencing. Pathogens.

[39] Wang Y., Sapula S.A., Whittall J.J., Blaikie J.M., Lomovskaya O., Venter H. (2024). Identification and characterization of *CIM-1*, a carbapenemase that adds to the family of resistance factors against last resort antibiotics. Commun. Biol..

[40] Schultz E., Barraud O., Madec J.Y., Haenni M., Cloeckaert A., Ploy M.C., Doublet B. (2017). Multidrug Resistance *Salmonella* Genomic Island 1 in a *Morganella morganii* subsp. *morganii* Human Clinical Isolate from France. mSphere.

[41] Bellais S., Poirel L., Leotard S., Naas T., Nordmann P. (2000). Genetic diversity of carbapenem-hydrolyzing metallo-beta-lactamases from *Chryseobacterium* (*Flavobacterium*) *indologenes*. Antimicrob. Agents Chemother..

[42] Han D., Ma S., He C., Yang Y., Li P., Lu L. (2024). Unveiling the genetic architecture and transmission dynamics of a novel multidrug-resistant plasmid harboring bla_NDM-5_ in *E. Coli* ST167: Implications for antibiotic resistance management. BMC Microbiol..

[43] Dutta C., Pan A. (2002). Horizontal gene transfer and bacterial diversity. J. Biosci..

[44] Mussi M.A., Limansky A.S., Relling V., Ravasi P., Arakaki A., Actis L.A., Viale A.M. (2011). Horizontal gene transfer and assortative recombination within the *Acinetobacter baumannii* clinical population provide genetic diversity at the single *carO* gene, encoding a major outer membrane protein channel. J. Bacteriol..

[45] Karampatakis T., Tsergouli K., Behzadi P. (2025). Carbapenem-Resistant *Pseudomonas aeruginosa’s* Resistome: Pan-Genomic Plasticity, the Impact of Transposable Elements and Jumping Genes. Antibiotics.

[46] Carraro N., Burrus V. (2015). The dualistic nature of integrative and conjugative elements. Mob. Genet. Elem..

[47] Wozniak R.A., Waldor M.K. (2009). A toxin-antitoxin system promotes the maintenance of an integrative conjugative element. PLoS Genet..

[48] Guglielmini J., Quintais L., Garcillan-Barcia M.P., De La Cruz F., Rocha E.P. (2011). The repertoire of ICE in prokaryotes underscores the unity, diversity, and ubiquity of conjugation. PLoS Genet..

[49] Burrus V., Waldor M.K. (2004). Shaping bacterial genomes with integrative and conjugative elements. Res. Microbiol..

[50] Andreu N., Zelmer A., Wiles S. (2011). Noninvasive biophotonic imaging for studies of infectious disease. FEMS Microbiol. Rev..

[51] Sentchilo V., Ravatn R., Werlen C., Zehnder A.J., van der Meer J.R. (2003). Unusual integrase gene expression on the *clc* genomic island in *Pseudomonas* sp. strain B13. J. Bacteriol..

[52] Wozniak R.A., Waldor M.K. (2010). Integrative and conjugative elements: Mosaic mobile genetic elements enabling dynamic lateral gene flow. Nat. Rev. Microbiol..

[53] Juhas M., van der Meer J.R., Gaillard M., Harding R.M., Hood D.W., Crook D.W. (2009). Genomic islands: Tools of bacterial horizontal gene transfer and evolution. FEMS Microbiol. Rev..

[54] Schmidt H., Hensel M. (2006). Pathogenicity islands in bacterial pathogenesis. Clin. Microbiol. Rev..

[55] Bellanger X., Payot S., Leblond-Bourget N., Guédon G. (2014). Conjugative and mobilizable genomic islands in bacteria: Evolution and diversity. FEMS Microbiol. Rev..

[56] Mingoia M., Morici E., Brenciani A., Giovanetti E., Varaldo P.E. (2015). Genetic basis of the association of resistance genes *mef(I)* (macrolides) and *catQ* (chloramphenicol) in streptococci. Front. Microbiol..

[57] Hensel M. (2004). Evolution of pathogenicity islands of *Salmonella enterica*. Int. J. Med. Microbiol..

[58] Partridge S.R., Kwong S.M., Firth N., Jensen S.O. (2018). Mobile Genetic Elements Associated with Antimicrobial Resistance. Clin. Microbiol. Rev..

[59] Stokes H.W., Gillings M.R. (2011). Gene flow, mobile genetic elements and the recruitment of antibiotic resistance genes into Gram-negative pathogens. FEMS Microbiol. Rev..

[60] San Millan A. (2018). Evolution of Plasmid-Mediated Antibiotic Resistance in the Clinical Context. Trends Microbiol..

[61] Siguier P., Perochon J., Lestrade L., Mahillon J., Chandler M. (2006). ISfinder: The reference centre for bacterial insertion sequences. Nucleic Acids Res..

[62] Johnson C.M., Grossman A.D. (2015). Integrative and Conjugative Elements (ICEs): What They Do and How They Work. Annu. Rev. Genet..

[63] Andersson D.I., Hughes D. (2010). Antibiotic resistance and its cost: Is it possible to reverse resistance?. Nat. Rev. Microbiol..

[64] van Houte S., Ekroth A.K., Broniewski J.M. (2016). The diversity-generating benefits of a prokaryotic adaptive immune system. Nature.

[65] Westra E.R., Buckling A., Fineran P.C. (2014). CRISPR-Cas systems: Beyond adaptive immunity. Nat. Rev. Microbiol..

[66] Domingues S., Harms K., Fricke W.F. (2012). Natural transformation facilitates transfer of transposons, integrons and gene cassettes between bacterial species. PLoS Pathog..

[67] Li X.Z., Plésiat P., Nikaido H. (2015). The challenge of efflux-mediated antibiotic resistance in Gram-negative bacteria. Clin. Microbiol. Rev..

[68] Munita J.M., Arias C.A. (2016). Mechanisms of Antibiotic Resistance. Microbiol. Spectr..

[69] Johnson J.S., Spakowicz D.J., Hong B.-Y., Petersen L.M., Demkowicz P., Chen L., Leopold S.R., Hanson B.M., Agresta H.O., Gerstein M. (2019). Evaluation of 16S rRNA gene sequencing for species and strain-level microbiome analysis. Nat. Commun..

[70] Magiorakos A.P., Srinivasan A., Carey R.B., Carmeli Y., Falagas M.E., Giske C.G., Harbarth S., Hindler J.F., Kahlmeter G., Olsson-Liljequist B. (2012). Multidrug-resistant, extensively drug-resistant and pandrug-resistant bacteria: An international expert proposal for interim standard definitions for acquired resistance. Clin. Microbiol. Infect..

[71] De Maio N., Shaw L.P., Hubbard A., George S., Sanderson N.D., Swann J., Wick R., AbuOun M., Stubberfield E., Hoosdally S.J. (2019). Comparison of long-read sequencing technologies in the hybrid assembly of complex bacterial genomes. Microb. Genom..

[72] Modi A., Vai S., Caramelli D., Lari M. (2021). The Illumina Sequencing Protocol and the NovaSeq 6000 System. Methods Mol. Biol..

[73] Wang Y., Zhao Y., Bollas A., Wang Y., Au K.F. (2021). Nanopore sequencing technology, bioinformatics and applications. Nat. Biotechnol..

[74] Wick R.R., Judd L.M., Gorrie C.L., Holt K.E. (2017). Unicycler: Resolving bacterial genome assemblies from short and long sequencing reads. PLoS Comput. Biol..

[75] Wick R.R., Judd L.M., Gorrie C.L., Holt K.E. (2017). Completing bacterial genome assemblies with multiplex MinION sequencing. Microb. Genom..

[76] Antipov D., Korobeynikov A., McLean J.S., Pevzner P.A. (2016). hybridSPAdes: An algorithm for hybrid assembly of short and long reads. Bioinformatics.

[77] Li H. (2016). Minimap and miniasm: Fast mapping and de novo assembly for noisy long sequences. Bioinformatics.

[78] Vaser R., Sovic I., Nagarajan N., Sikic M. (2017). Fast and accurate de novo genome assembly from long uncorrected reads. Genome Res..

[79] Seemann T. (2014). Prokka: Rapid prokaryotic genome annotation. Bioinformatics.

[80] Chklovski A., Parks D.H., Woodcroft B.J., Tyson G.W. (2023). CheckM2: A rapid, scalable and accurate tool for assessing microbial genome quality using machine learning. Nat. Methods.

[81] Galperin M.Y., Makarova K.S., Wolf Y.I., Koonin E.V. (2015). Expanded microbial genome coverage and improved protein family annotation in the COG database. Nucleic Acids Res..

[82] Aramaki T., Blanc-Mathieu R., Endo H., Ohkubo K., Kanehisa M., Goto S., Ogata H. (2020). KofamKOALA: KEGG Ortholog assignment based on profile HMM and adaptive score threshold. Bioinformatics.

[83] Robinson J.T., Thorvaldsdóttir H., Winckler W., Guttman M., Lander E.S., Getz G., Mesirov J.P. (2011). Integrative genomics viewer. Nat. Biotechnol..

[84] Minh B.Q., Schmidt H.A., Chernomor O., Schrempf D., Woodhams M.D., von Haeseler A., Lanfear R. (2020). IQ-TREE 2: New Models and Efficient Methods for Phylogenetic Inference in the Genomic Era. Mol. Biol. Evol..

[85] Kalyaanamoorthy S., Minh B.Q., Wong T.K.F., von Haeseler A., Jermiin L.S. (2017). ModelFinder: Fast model selection for accurate phylogenetic estimates. Nat. Methods.

[86] Eren A.M., Esen Ö.C., Quince C., Vineis J.H., Morrison H.G., Sogin M.L., Delmont T.O. (2015). Anvi’o: An advanced analysis and visualization platform for ‘omics data. PeerJ.

[87] Hyatt D., Chen G.L., Locascio P.F., Land M.L., Larimer F.W., Hauser L.J. (2010). Prodigal: Prokaryotic gene recognition and translation initiation site identification. BMC Bioinform..

[88] Yoon B.J. (2009). Hidden Markov Models and their Applications in Biological Sequence Analysis. Curr. Genom..

[89] Babicki S., Arndt D., Marcu A., Liang Y., Grant J.R., Maciejewski A., Wishart D.S. (2016). Heatmapper: Web-enabled heat mapping for all. Nucleic Acids Res..

[90] Alcock B.P., Huynh W., Chalil R., Smith K.W., Raphenya A.R., Wlodarski M.A., Edalatmand A., Petkau A., Syed S.A., Tsang K.K. (2023). CARD 2023: Expanded curation, support for machine learning, and resistome prediction at the Comprehensive Antibiotic Resistance Database. Nucleic Acids Res..

[91] Bortolaia V., Kaas R.S., Ruppe E., Roberts M.C., Schwarz S., Cattoir V., Philippon A., Allesoe R.L., Rebelo A.R., Florensa A.F. (2020). ResFinder 4.0 for Predictions of Phenotypes from Genotypes. J. Antimicrob. Chemother..

[92] Camacho C., Coulouris G., Avagyan V., Ma N., Papadopoulos J., Bealer K., Madden T.L. (2009). BLAST+: Architecture and Applications. BMC Bioinform..

[93] Gupta S.K., Padmanabhan B.R., Diene S.M., Lopez-Rojas R., Kempf M., Landraud L., Rolain J.M. (2014). ARG-ANNOT, a New Bioinformatic Tool to Discover Antibiotic Resistance Genes in Bacterial Genomes. Antimicrob. Agents Chemother..

[94] Grant J.R., Enns E., Marinier E., Mandal A., Herman E.K., Chen C.Y., Graham M., Van Domselaar G., Stothard P. (2023). Proksee: In-depth characterization and visualization of bacterial genomes. Nucleic Acids Res..

[95] Chen L., Yang J., Yu J., Yao Z., Sun L., Shen Y., Jin Q. (2005). VFDB: A Reference Database for Bacterial Virulence Factors. Nucleic Acids Res..

[96] Bertelli C., Laird M.R., Williams K.P., Lau B.Y., Hoad G., Winsor G.L., Brinkman F.S., Simon Fraser University Research Computing Group (2017). IslandViewer 4: Expanded Prediction of Genomic Islands for Larger-Scale Datasets. Nucleic Acids Res..

[97] Néron B., Littner E., Haudiquet M., Perrin A., Cury J., Rocha E.P.C. (2022). IntegronFinder 2.0: Identification and Analysis of Integrons across Bacteria, with a Focus on Antibiotic Resistance in Klebsiella. Microorganisms.

[98] Carattoli A., Hasman H. (2020). PlasmidFinder and In Silico pMLST: Identification and Typing of Plasmid Replicons in Whole-Genome Sequencing (WGS). Methods Mol. Biol..

