# Supplementary material for: Genomic Insights into Emerging Multidrug-Resistant Chryseobacterium indologenes Strains: First Report from Thailand
Source: Antibiotics (Basel). 2025 Jul 24;14(8):746. doi: 10.3390/antibiotics14080746 (PMC12382657; doi:10.3390/antibiotics14080746)
Supplement: Supplementary file 1 [file antibiotics-14-00746-s001.zip › antibiotics-3752539-supplementary.pdf]

Supplementary

Table S1. Demographic and clinical characteristics of *C. indologenes* infection

Clinical characteristics	No. of patients (n = 12)	%
**Age ***		
Range	44 - 82	
Mean ± SD	63.33 (±13.05)	
**Gender ***: Male	10	83.33
**Primary diagnosis**	10	83.33
Pneumonia	3	30.0
Malignancy	2	20.0
Respiratory diseases	2	20.0
Cirrhosis of liver	1	10.0
Urinary tract infection	1	10.0
Fever	1	10.0
**Comorbidities**	9	75.0
Anaemia	5	55.55
Acute renal failure	5	55.55
Essential hypertension	4	44.44
Septic shock & Septicaemia	4	44.44
Chronic kidney disease	3	33.33
Chronic viral hepatitis B	3	33.33
Cerebral infarction	3	33.33
Type 2 diabetes mellitus	3	33.33
Cirrhosis of lung	2	22.22
COVID-19	1	11.11
No co-morbidity	1	10.0
**Hospitalized patients**	9	75
**OPD patient**	1	8.33
**Hospital stay duration**		
Days until infection, Mean (± SD)	13.30 (±7.69)	
Total duration, Mean (± SD)	17.70 (±13.44)	
**Wards**		
Internal medicine wards	6	66.67
Surgery ward	1	11.11
Cohort ward	1	11.11
Private room	1	11.11
Others	2	22.22
**ICUs**	3	33.33
**Dead**	2	20
**Specimens**		
Urine	10	83.33
Sputum	2	16.67
**Co-infection with other organisms ***	5	41.67
**Invasive procedures**	9	90.0
Catheter	9	90.0
Urine catheter	7	70.0
Respirator	7	70.0
Gastric tube	5	50.0
**Predisposing factors**	9	90.0
Surgery	3	30.0
Chemo/radio therapy	2	20.0
Dialysis	2	20.0
**No data**	2	16.67

* Age, Gender, and Co-infection could be calculated from all patients (n = 12), while other data could be accessed from 10 patients.; SD, Standard Deviation; OPD, Out-Patient Department; ICUs, Intensive care units

Figure S1. Antibiotic susceptibility and interpretation of 12 *C. indologenes* isolates

Drug group	Penicillin	3rd Gen. Cephalosporins			4th Gen. Cephalosporin	Carbapenems		Aminoglycosides		Fluoroquinolones		Sulfonamide	Number of resistant drugs per isolate (n = 12)	Number of resistant drug groups per isolate (n = 6)	Phenotype
Antibiotic / Isolate	Piperacillin/ Tazobactam	Ceftazidime	Ceftriaxone	Cefotaxime	Cefepime	Imipenem	Meropenem	Amikacin	Gentamicin	Ciprofloxacin	Levofloxacin	Trimethoprim/ Sulfamethoxazole			
CMCI01	>64 (R)	>32 (R)	>32 (R)	>32 (R)	>32 (R)	>16 (R)	>16 (R)	>32 (R)	>8 (R)	>2 (R)	>8 (R)	≤1 (S)	11	5	XDR
CMCI05	>64 (R)	>32 (R)	>32 (R)	>32 (R)	>32 (R)	>16 (R)	>16 (R)	>32 (R)	>8 (R)	>2 (R)	>8 (R)	≤1 (S)	11	5	XDR
CMCI10	>64 (R)	>32 (R)	>32 (R)	>32 (R)	>32 (R)	>16 (R)	>16 (R)	>32 (R)	>8 (R)	>2 (R)	>8 (R)	≤1 (S)	11	5	XDR
CMCI11	>64 (R)	>32 (R)	>32 (R)	>32 (R)	>32 (R)	>16 (R)	>16 (R)	>32 (R)	>8 (R)	>2 (R)	>8 (R)	≤1 (S)	11	5	XDR
CMCI12	>64 (R)	>32 (R)	>32 (R)	>32 (R)	>32 (R)	>16 (R)	>16 (R)	8 (S)	>8 (R)	>2 (R)	>8 (R)	≤1 (S)	10	5	XDR
CMCI13	>64 (R)	16 (I)	32 (I)	>32 (R)	2 (S)	>16 (R)	>16 (R)	32 (I)	8 (I)	1 (S)	0.5 (S)	≤1 (S)	8	3	MDR
CMCI14	>64 (R)	>32 (R)	>32 (R)	>32 (R)	>32 (R)	>16 (R)	>16 (R)	>32 (R)	>8 (R)	>2 (R)	>8 (R)	≤1 (S)	11	5	XDR
CMCI23	>64 (R)	>32 (R)	>32 (R)	>32 (R)	>32 (R)	>16 (R)	>16 (R)	>32 (R)	>8 (R)	>2 (R)	>8 (R)	≤1 (S)	11	5	XDR
CMCI46	>64 (R)	>32 (R)	>32 (R)	>32 (R)	>32 (R)	>16 (R)	>16 (R)	>32 (R)	>8 (R)	>2 (R)	>8 (R)	≤1 (S)	11	5	XDR
CMCI56	>64 (R)	>32 (R)	>32 (R)	>32 (R)	>32 (R)	>16 (R)	>16 (R)	>32 (R)	>8 (R)	>2 (R)	>8 (R)	≤1 (S)	11	5	XDR
CMCI60	>64 (R)	>32 (R)	>32 (R)	>32 (R)	>32 (R)	>16 (R)	>16 (R)	>32 (R)	>8 (R)	>2 (R)	>8 (R)	≤1 (S)	11	5	XDR
CMCI63	>64 (R)	>32 (R)	>32 (R)	>32 (R)	>32 (R)	>16 (R)	>16 (R)	>32 (R)	>8 (R)	>2 (R)	>8 (R)	≤1 (S)	11	5	XDR
Number of isolate															
S	0	0	0	0	1	0	0	1	0	1	1	12			
I	0	1	1	0	0	0	0	1	1	0	0	0			
R	12	11	11	12	11	12	12	10	11	11	11	0			
Break points															
S	≤ 16/4	≤ 8	≤ 8	≤ 8	≤ 8	≤ 4	≤ 4	≤ 16	≤ 4	≤ 1	≤ 2	≤ 2/38			
I	32/4–64/4	16	16-32	16-32	16	8	8	32	8	2	4	-			
R	≥ 128/4	≥ 32	≥ 64	≥ 64	≥ 32	≥ 16	≥ 16	≥ 64	≥ 16	≥ 4	≥ 8	≥ 4/76			

3rd and 4th gen., Third and Fourth generation of cephalosporins; S, susceptible; I, intermediate resistant; R, resistant; ≤, Less than or equal to; >, Greater than MDR (Multidrug resistance), Isolates resistant to at least one agent in three or more antibiotic group.; XDR (Extensively Drug Resistance), Isolates resistant to at least one drug in all but two or fewer antibiotic group.

Table S2. SNP variances of 12 *C. indologenes* isolates

Variant type	Number of SNP position											
	CMCI 01	CMCI 05	CMCI 10	CMCI 11	CMCI 12	CMCI 13	CMCI 14	CMCI 23	CMCI 46	CMCI 56	CMCI 60	CMCI 63
Frameshift	88	91	92	91	87	87	87	88	87	85	84	84
Missense	5863	5862	5860	5863	5667	5734	5840	5859	5474	5467	5463	5451
Intragenic	8	8	8	8	8	8	8	8	8	8	8	8
Disruptive in-frame insertion/deletion	14	14	14	14	14	16	14	14	15	15	15	15
Conservative in-frame insertion/deletion	11	11	11	11	12	10	10	12	10	12	11	11
Gene fusion	1	1	1	1	1	1	1	1	1	0	0	0
Initiator codon variant	3	3	3	3	3	2	3	3	2	3	3	2
Non-coding transcript	6	6	6	6	6	6	6	6	5	4	5	5
Start & Stop & Splice region	67	69	63	67	65	58	68	68	36	35	37	35
synonymous	20971	20931	20973	20954	20411	20760	20890	20961	13746	13745	13725	13748
Total	27032	26996	27031	27018	26274	26682	26927	27020	19384	19374	19351	19359

SNP, single nucleotide polymorphism

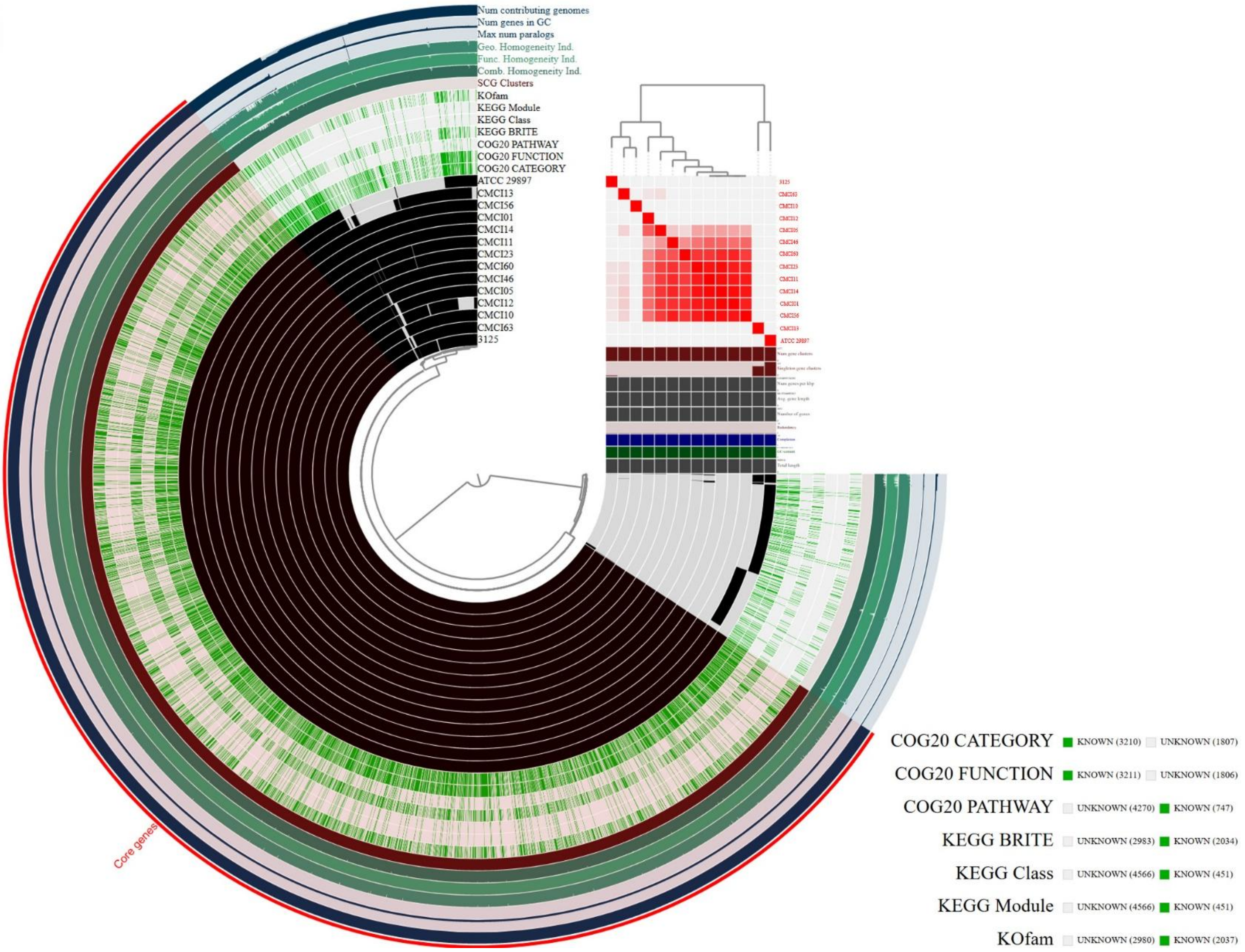

Figure S2. Pangenome analysis of *C. indologenes* isolates. The whole genomes of 12 *C. indologenes* strains were compared with two reference strains of *C. indologenes*, ATCC 29897 and 3125. The comparison is based on the presence or absence of gene clusters across the analyzed genomes, with the circular diagram divided into three main sections. The innermost section displays gene presence in solid color and absence in gray, with core genes (present in 100% of the compared genomes) highlighted in red. The second section details single-copy gene clusters, such as KOfam, KEGG modules, and COG20 categories, with known genes shown in green and unknown genes in gray. The third section presents genome properties, including the homogeneity index, contributing genomes, number of genes per gene cluster, and maximum number of paralogs. A heatmap illustrates the Average Nucleotide Identity (ANI) among all 14 isolates, where darker colors indicate higher identity and lighter colors indicate lower identity. The dendrogram within the circular diagram shows gene clustering relationships based on Euclidean distance and Ward linkage.

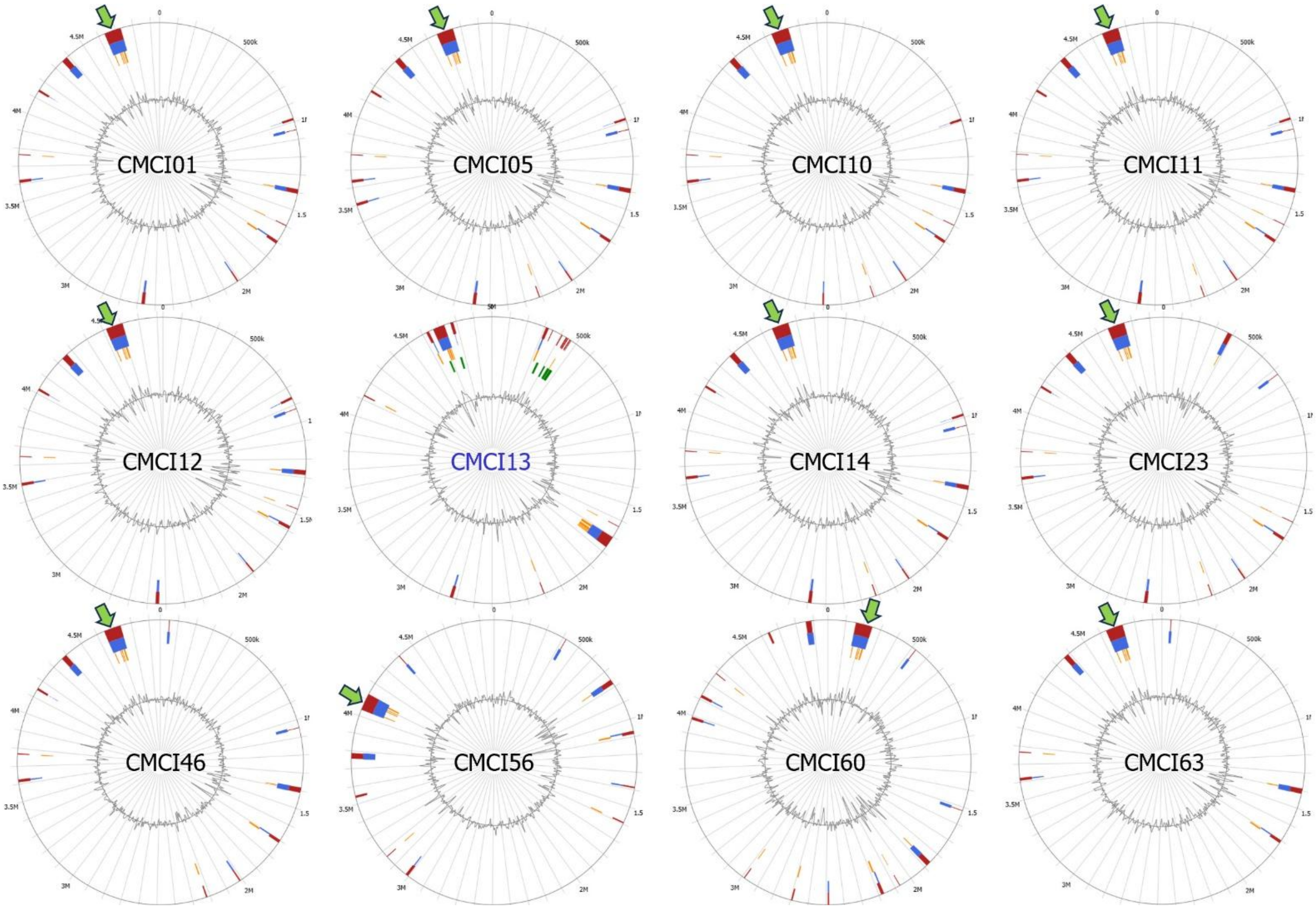

Figure S3. Comparison of genomic island patterns within 12 *C. indologenes* chromosomes. Genomic island was predicted by IslandViewer4. The colored blocks showed GIs, corresponding to the prediction method used. IslandPick (green), IslandPath-DIMOB (blue), SIGI-HMM (orange), and the integrated findings are displayed in dark red. The largest GIs were highlight with green arrow.
